# The Consistency of Gastropod Identified Neurons Distinguishes Intra-Individual Plasticity From Inter-Individual Variability in Neural Circuits

**DOI:** 10.3389/fnbeh.2022.855235

**Published:** 2022-03-03

**Authors:** Arianna N. Tamvacakis, Joshua L. Lillvis, Akira Sakurai, Paul S. Katz

**Affiliations:** ^1^Department of Biology, University of Arkansas, Fayetteville, AR, United States; ^2^Howard Hughes Medical Institute, Ashburn, VA, United States; ^3^Neuroscience Institute, Georgia State University, Atlanta, GA, United States; ^4^Department of Biology, University of Massachusetts Amherst, Amherst, MA, United States

**Keywords:** injury, neuromodulation, RNA sequencing (RNA-seq), nudibranch behavior, electrophysiology, species differences, individual variability

## Abstract

Gastropod mollusks are known for their large, individually identifiable neurons, which are amenable to long-term intracellular recordings that can be repeated from animal to animal. The constancy of individual neurons can help distinguish state-dependent or temporal variation within an individual from actual variability between individual animals. Investigations into the circuitry underlying rhythmic swimming movements of the gastropod species, *Tritonia exsulans* and *Pleurobranchaea californica* have uncovered intra- and inter-individual variability in synaptic connectivity and serotonergic neuromodulation. *Tritonia* has a reliably evoked escape swim behavior that is produced by a central pattern generator (CPG) composed of a small number of identifiable neurons. There is apparent individual variability in some of the connections between neurons that is inconsequential for the production of the swim behavior under normal conditions, but determines whether that individual can swim following a neural lesion. Serotonergic neuromodulation of synaptic strength intrinsic to the CPG creates neural circuit plasticity within an individual and contributes to reorganization of the network during recovery from injury and during learning. In *Pleurobranchaea*, variability over time in the modulatory actions of serotonin and in expression of serotonin receptor genes in an identified neuron directly reflects variation in swimming behavior. Tracking behavior and electrophysiology over hours to days was necessary to identify the functional consequences of these intra-individual, time-dependent variations. This work demonstrates the importance of unambiguous neuron identification, properly assessing the animal and network states, and tracking behavior and physiology over time to distinguish plasticity within the same animal at different times from variability across individual animals.

## Introduction

“*Unexplained variation in behavior is weak evidence for noisy indeterminacy but strong evidence for unknown modulating factors.*”

– Theodore Bullock (2000)

A goal of studying behavioral variability is to find the source of that variability in the neural circuits that control the behavior. Just as there can be individual differences in behavior, neural circuits can also exhibit individual differences. Even the simplest circuit contains a myriad of physiological and molecular components that are each subject to variability (Goaillard et al., 2009; Marder, 2011). Finding the “unknown modulating factors” in Bullock’s words, can lead to a deeper mechanistic understanding of the function of the circuits. However, determining whether there are individual differences in neural circuitry is more challenging than noting differences in behavior because it requires making repeated measurements from the same circuit elements in multiple individuals. Having reliably identifiable neurons and synapses is required to distinguish whether those circuit components vary between individuals or if they are variable over time within an individual.

The nervous systems of gastropods, arthropods, annelids, and nematodes are well-suited for such repeated measurements because they contain individually identifiable neurons (Hoyle, 1975; Leonard, 2000; Brodfuehrer and Thorogood, 2001; Comer and Robertson, 2001; Katz and Quinlan, 2018). The size, number, location, anatomy, and neurochemistry of individual neurons are stereotyped among members of the same species, allowing the neural mechanisms underlying behaviors in some of these animals to be determined using multiple intracellular microelectrode recordings. The neurons are large and resist damage from multiple microelectrode penetrations, facilitating hours-long recordings and even multiple recordings of the same neuron over a course of days. The clear-cut identification of neurons also allows hundreds of recordings to be made from the same neuron in different animals.

Ironically, it is the consistency of the neurons that allows individual differences in neural circuits to be revealed; the identities of the individual neurons are so unambiguous that variations in their properties or synapses do not cause them to be mistaken for a different neuron. Furthermore, the presence of neurons is so highly conserved that the characteristics that are used for identification of a neuron from animal to animal in one species can be used to recognize the same neuron in other species (Croll, 1987; Newcomb et al., 2012). This allows the properties and connectivity of individual neurons to be compared across species, providing the opportunity for natural experiments regarding the functional significance of individual variation.

In this review, we highlight examples of intra- and inter-individual variabilities from the central pattern generator (CPG) circuits underlying swimming in two sea slugs, the nudibranch, *Tritonia exsulans* (formerly *Tritonia diomedea*), and the pleurobranchomorph, *Pleurobranchaea californica*. The work shows that differences that could be mistaken for variation between individuals can be attributed to differences in state of the neurons and synapses over time within an individual. There are also individual differences in the circuits that have no consequence for behavior under normal circumstances but affect the susceptibility of the circuit to a lesion. Without the consistency of neural identification and the ability to monitor neurons over several days, individual differences may appear as “noisy indeterminacy,” rather than having causal factors that vary within an individual over time.

## A Brief History of Research on Variability in Invertebrate Neural Circuits

An early strategy employed to study the neural basis of behavior was to focus on behaviors that showed little or no variability, including rhythmic motor patterns produced by CPG circuits (Getting, 1986; Marder and Calabrese, 1996; Marder and Bucher, 2001). However, one of the principles that arose from this work is that even a simple, anatomically defined network can produce a variety of different motor patterns as a result of the neuromodulatory actions of amines and peptides. Amines, such as serotonin (5-HT) can alter membrane conductances and synaptic properties to change the dynamics of the network on a moment-to-moment basis (Harris-Warrick and Marder, 1991). Thus, it became important to identify not only the neurons in the network, but the state of the network to determine the mechanisms underlying various forms of the rhythmic output.

Similarly, research on identified neurons in invertebrates showed that properties of neurons and synapses could be modified by the history of activity though the circuit, leading to various forms of learning and memory (Carew and Sahley, 1986; Menzel and Benjamin, 2013). Thus, any study of the neural basis of individual variability must also take into account the history of neural firing and the history of previous experience of the animal.

Finally, another realization from electrophysiological research on invertebrate neural circuits was that even though circuits are composed of a small number of identified neurons, there are still multiple mechanisms that could produce the same output (Prinz et al., 2004; Rodriguez et al., 2013; Marder et al., 2016). Moreover, individual identified neurons display variations in membrane properties that are not well described by the mean of the population, which makes it difficult to model the circuit (Golowasch et al., 2002). Furthermore, individual differences found in neural circuits do not necessarily translate to individual differences in behavior (Marder, 2011; Marder et al., 2015). Although individual differences in neural circuits may have no consequences for behavior under standard conditions, they might differentiate the behaviors of two individuals when challenged with extreme conditions or injury (Marder and Rue, 2021). Thus, it is important to consider a range of conditions when assessing the behavioral consequences of individual differences in circuit properties.

## Individually Identifiable Neurons Comprise the Circuit Underlying *Tritonia* Swimming

**FIGURE 1:**
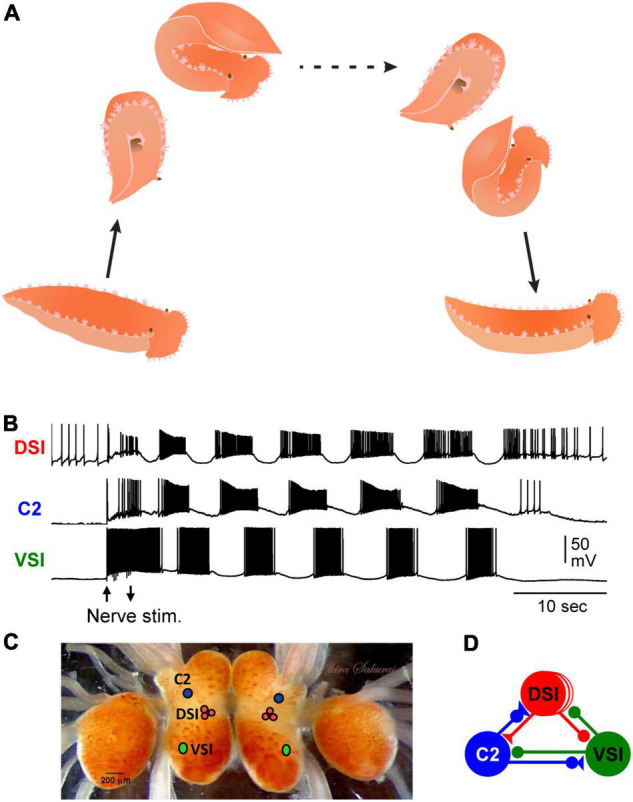
*Tritonia* swimming behavior and swim motor pattern mechanism. **(A)** Illustration of *Tritonia* dorsal-ventral flexion swimming behavior. **(B)** Simultaneous intracellular recordings from identified neurons, DSI, C2, and VSI during a swim motor pattern elicited by stimulation of a body wall nerve (arrows). **(C)** A photograph of the dorsal side of the *Tritonia* brain, showing the locations of the swim interneurons (C2, DSI, and VSI). **(D)** A schematic diagram of the swim CPG. Triangles represent excitatory synapses, circles represent inhibitory synapses, and combined represent multicomponent synapses.

The nudibranch, *Tritonia* provided one of the earliest examples of the roles of identified neurons in the production of behavior (Willows, 1967). The animal produces a stereotyped escape swimming behavior when attacked by a predatory sea star or when encountering a noxious stimulus (Willows and Hoyle, 1969). The escape swim response consists of a series of alternating dorsal and ventral whole-body flexions that lasts about 1 min (Figure 1A). The performance of the swim is robust: *Tritonia* reliably swims whenever it is stimulated.

A fictive swim motor pattern is reliably produced *ex vivo* by electrically stimulating a body wall nerve in an isolated brain preparation, allowing the neural basis for the stereotyped behavior to be studied (Figure 1B) (Katz, 2009; Katz and Sakurai, 2017). There are three bilaterally represented neuron types that form a CPG circuit (Figures 1C,D), which produces the bursting activity underlying the production of the rhythmic dorsal-ventral swim behavior. The three CPG neurons are: ventral swim interneuron-B (VSI), cerebral neuron 2 (C2), and three serotonergic dorsal swim interneurons (DSIs) (Getting, 1989a; Katz, 2009). Each neuron type is uniquely identifiable by its soma position, neuroanatomy, neurotransmitter phenotype, and activity pattern during the swim motor pattern (Figures 1C,D). The monosynaptic connections between these neurons have been determined using pair-wise intracellular microelectrode recordings (Getting, 1981). Modeling the properties of the neurons and their synaptic connectivity showed them to be sufficient to cause the rhythmic bursting pattern (Getting, 1989b). Thus, the *Tritonia* swim motor pattern and its neurons are consistent, allowing investigations into the presence and functional significance of variations.

## Functional Consequences of Individual Differences in Synaptic Connections Are Revealed by Neural Injury

As has been noted in other systems, the strengths of synapses between any particular pair of neurons can be highly variant with little or no effect on the behavioral output of the circuit (Goaillard et al., 2009; Roffman et al., 2012). Theoretically, it is understood that a fixed network topology may still have many solutions to produce the same output (Prinz et al., 2004; Onasch and Gjorgjieva, 2020). Although circuit variation across individuals may have no effect under “normal conditions,” behavioral differences might emerge when the system is challenged by environmental changes (Marder and Rue, 2021).

**FIGURE 2:**
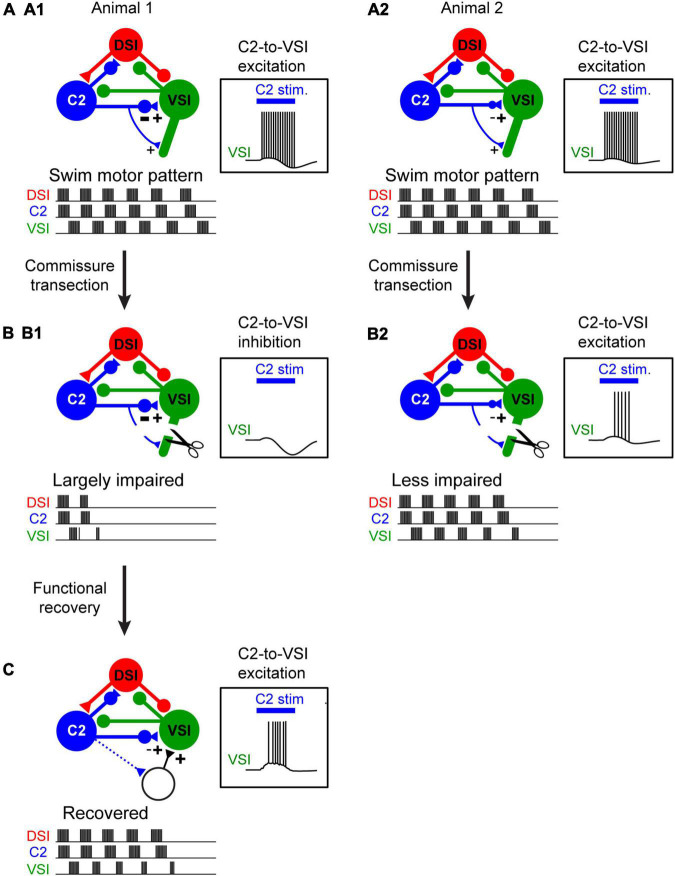
The extent of injury-induced impairment in the swim motor pattern depends on the strength of a particular synapse. **(A1,A2)** Schematic illustration of differences in synaptic strength and motor output under normal conditions. Animal 1 (A1) and Animal 2 (A2) exhibit no apparent difference in the swim motor pattern. C2 synapses on VSI proximally and distally. The two animals differ in the strength of the proximal inhibition. But this has no effect on the ability of C2 stimulation (blue bar) to elicit a spike train in VSI. **(B1,B2)** When the distal synapse from C2 to DSI is cut, the motor pattern in Animal 1 is impaired because the proximal synapse had a strong inhibitory component and C2 fails to excite VSI (B1). However, the motor pattern in Animal 2 is less impaired because the proximal synapse was less inhibitory so C2 continues to excite VSI (B2). **(C)** Injury-induced loss of swim motor pattern is restored within a few hours by the recruitment of unidentified neurons with excitatory synapses to VSI (dotted lines). This recovery also involves the serotonergic DSIs (not illustrated).

Synapses in the *Tritonia* swim CPG show variation that does not have an effect on the motor pattern in a normal intact system, but causes individual animals to differ in their susceptibility to a midline lesion of the nervous system (Sakurai et al., 2014). Cutting the pedal commissure, which contains the axons of all three CPG neurons, disables swimming behavior in approximately half of the animals tested (Sakurai and Katz, 2009a). Similarly, about half of the isolated brain preparations fail to produce a swim motor pattern after the commissure is cut or action potential propagation is blocked (Figures 2A,B). Individual differences in the strength of the inhibitory synapse from C2 to VSI at the time of the lesion cause the differences in susceptibility (Sakurai et al., 2014). Under normal conditions, variation in the strength of this synapse has no effect on the swim motor pattern, but animals with a larger inhibitory component are susceptible to having the motor pattern fail after lesion.

There are also individual differences in recovery from this lesion (Sakurai and Katz, 2009a). The mechanism of recovery involves a reorganization of the CPG through recruitment of additional neurons and involves the activity of the serotonergic DSIs (Figure 2C). The extent of recovery was correlated with the change in the depolarization in VSI caused by stimulating DSI and C2 together (Sakurai et al., 2016), implying that serotonergic modulation is involved in the recovery through an unknown mechanism. Neuromodulatory mechanisms have been implicated in recovery from injury in several invertebrate CPG networks across phyla (Puhl et al., 2018; Golowasch, 2019). A connection between injury responses and serotonergic neuromodulation has been proposed based on research in *Aplysia* (Walters and Ambron, 1995). Serotonin also has been implicated in recovery from spinal cord injury (Ghosh and Pearse, 2014; Huang et al., 2021). Although these types of lesions are not likely to occur under natural conditions, the plasticity itself is present and may play a role in maintaining circuit function over the lifetime of an animal.

The injury studies reveal that there can be variation in the system that normally is of no consequence to the behavior. Such hidden variation and its consequences would not have been revealed without the ability to monitor activity from the same neurons over days. The question arises as to whether the hidden differences that were identified are “noisy indeterminacy” or whether the apparent individual differences in synapses reflect the history of the animal and thus may be an intra-animal difference that emerged over time.

## Variability in Neuromodulatory Actions Caused by Synaptic State-Dependence

Neuromodulation is a means to achieve behavioral flexibility in neural circuits within an individual. It allows a structurally stable circuit to produce different patterns of activity by altering membrane and synaptic conductances (Katz and Calin-Jageman, 2008; Marder et al., 2014). Serotonergic neuromodulation alters motor patterns (Katz, 1995), modifies sensory responses (Sizemore et al., 2020), changes responses to social interactions (Cattaert et al., 2010), mediates learned responses (Brunelli et al., 1976; Jacobs and Gelperin, 1981), and plays a role in recovery from injury (Husch et al., 2012; Ghosh and Pearse, 2014). Neuromodulation has also been noted to be a source of variability between animals (Maloney, 2021). This occurs both in invertebrate circuits with identified neurons such as the stomatogastric ganglion of crabs (Hamood and Marder, 2014), but also in vertebrates, which are not constrained in the same way by the small number of neurons (Parker and Bevan, 2006; Sharples and Whelan, 2017). For example, in zebrafish, variations in serotonergic Raphe neurons cause individual differences in habituation of the acoustic startle response (Pantoja et al., 2016).

In the *Tritonia* swim circuit, serotonin plays an intrinsic modulatory role; it is released from the DSI and enhances the strength of synapses made by the other CPG neurons C2 and VSI (Katz et al., 1994; Katz and Frost, 1996; Sakurai and Katz, 2003). Computer simulations suggest that this neuromodulatory action is necessary for the network of neurons to produce its rhythmic pattern of activity (Calin-Jageman et al., 2007).

**FIGURE 3:**
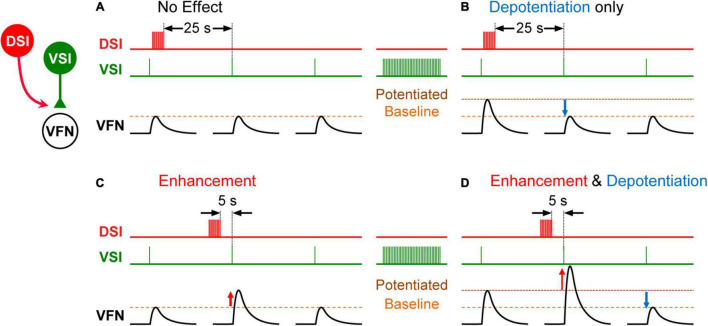
Schematic representation showing state- and timing-dependent neuromodulation of synaptic strength by a serotonergic DSI neuron. **(A)** When the strength of the VSI-to-VFN synapse is at baseline, a DSI spike train delivered 25 s before the next VSI spike has no effect on the synaptic strength. **(B)** After a VSI spike train, the VSI synapse shows post-tetanic potentiation. When potentiated, a DSI spike train delivered 25 s before the next VSI spike depotentiates the synapse. **(C)** When the strength of the VSI-to-VFN synapse was at baseline, a DSI spike train delivered 5 s prior to the next VSI spike produces transient heterosynaptic enhancement (upward red arrow), which lasts for about 15 s. **(D)** When potentiated, a DSI spike train delivered 5 s prior to the next VSI spike produces transient enhancement and a subsequent depotentiation of the synapse. DSI spike train (red trace), VSI spikes (green trace), and the synaptic potentials in VFN (black traces) are shown in each. In each trace, single VSI spikes each produce EPSPs in the postsynaptic VFN neuron. Changes in the synaptic strength are indicated by dashed lines (baseline, potentiated).

The effect of exogenous serotonin on VSI-evoked synaptic potentials was found to vary from individual to individual, sometimes potentiating the synapses and sometimes depressing them (Sakurai and Katz, 2003). The cause of this variability remained a mystery for almost 6 years until it was found that the action of serotonin and indeed the serotonergic DSI was dependent upon the firing history of the neurons that it was modulating (Sakurai and Katz, 2009b). VSI-evoked synaptic currents recorded in a ventral flexion neuron (VFN) exhibit their own homosynaptic potentiation (Figure 3); if VSI fires with a spike frequency of just 5 Hz for a few seconds, its synaptic output can increase up to twofold (Figure 3B). If a DSI is stimulated to release serotonin when VSI synapses are already potentiated, the serotonin causes the synapses to depotentiate (Figure 3B). Additional DSI stimulation has no further effect once the homosynaptic potentiation has been reversed. In addition, DSI heterosynaptically enhances VSI-evoked synaptic currents when stimulated shortly before VSI spikes, thereby increasing the VSI-evoked synaptic currents regardless of their potentiation state (Figures 3C,D; Sakurai and Katz, 2009b). In this case, the variability was not inter-individual, it was an intra-individual state- and timing-dependent effect.

## Variability in Behavior and Network Size Caused by Behavioral History

As it was necessary to know the state of individual neurons in order to assess the effects of neuromodulation, it also may be necessary to know the behavioral history of an individual animal to assess potential variability in how the network will respond to subsequent stimuli. In *Tritonia*, the strength of the swim response and size of the network underlying it vary depending on recent swim history. Although the *Tritonia* swim CPG may be consistent in its composition of neurons, the downstream elements that translate the rhythm into motor output vary. There are over 50 flexion neurons (FNs) that exhibit coordinated bursting that is driven by the CPG (Hume et al., 1982; Hume and Getting, 1982). A subset of FNs exhibit within-animal variability in their participation in the motor program from cycle-to-cycle and across swim episodes (Hill et al., 2012). This network variability may be reflective of some level of behavioral flexibility in this so-called fixed action pattern.

The *Tritonia* escape swim is a rare event in the animal’s life (Wyeth and Willows, 2006). An individual exhibits a consistent response when tested with a strong stimulus at long intervals. However, if stimulated a second time within 5 min, the swim response starts sooner and is more vigorous than after the first stimulus, indicating a form of sensitization (Frost et al., 1998; Hill et al., 2015). This sensitization is correlated with an increased participation of FNs. Stimulating the serotonergic DSIs also increases network size (Hill et al., 2015), plausibly by enhancing the synaptic strength of connections within the network. The participation of a subset of follower neurons is therefore not invariant, but a consequence of the history and activity of the CPG neurons.

## Variation in Behavior, Neuromodulation, and Gene Expression in *Pleurobranchaea*

**FIGURE 4:**
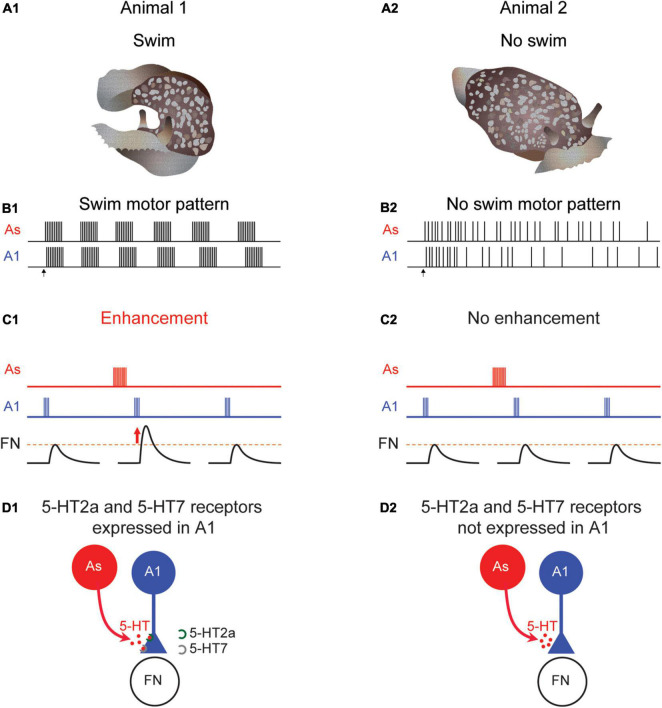
Differences in *Pleurobranchaea* swimming, neuromodulation, and serotonin receptor expression. When tested, some animals swim **(A1)** produce a rhythmic swim motor pattern **(B1)**. At other times, an animal might not swim **(A2)** and would not produce a swim motor pattern **(B2)**. When the animal swims the A1 to follower neuron (FN) synapse is strengthened following serotonergic As stimulation in a swimming animal **(C1)**. But at times when the animal does not swim, there is no enhancement **(C2)**. Serotonin receptor subtypes 5-HT2a and 5-HT7 are present in preparations that produce a swim motor pattern **(D1)** but not in ones that do not **(D2)**.

In contrast to *Tritonia*, there is a great deal of individual variability in the generation of a swim response in *Pleurobranchaea* (Figures 4A1,A2). On any given day, fewer than 30% of the individual animals respond to strong noxious stimulus with a rhythmic swimming response (Jing and Gillette, 1995; Lillvis and Katz, 2013). However, when tested on different days, the same animal shows different propensities to swim. Furthermore, even when dissected from the animal, the isolated brain is similarly variable in the production of a fictive swim motor pattern (Figures 4B1,B2), indicating that the cause of that variability is in the brain and not the periphery. Thus, the apparent individual variability in behavior is not caused by inherent differences between individuals but is most likely temporal variability of each individual.

The swim CPG in *Pleurobranchaea* contains identified neurons homologous to DSI and C2, known as the As and A1 neurons, respectively (Jing and Gillette, 1995, 1999; Lillvis et al., 2012; Newcomb et al., 2012). As with *Tritonia*, the DSI homolog (As) enhances the strength of synaptic potentials evoked by the C2 homolog (A1) (Figure 4C1). However, unlike in *Tritonia*, the neuromodulatory effect is sometimes absent; neither As stimulation nor serotonin application causes a change in the amplitude of A1-evoked synaptic potentials (Figure 4C2). This variation correlates with the swim motor pattern; preparations that do not produce a swim motor pattern, also do not exhibit serotonergic enhancement of A1-evoked synapses (Lillvis and Katz, 2013). Thus, in this case, variation in the response to serotonin may be the cause of variation in behavior.

The variation in serotonergic neuromodulation is mirrored by differences in the expression of particular serotonin receptors (5-HTRs) in A1. Plucking out the somata of individual A1 neurons from preparations that either did or did-not exhibit the swim motor pattern allowed for single-cell gene expression comparisons. Using single-cell RNA sequencing and single neuron quantitative PCR, Tamvacakis et al. (2018) found that A1 neurons from individual *Pleurobranchaea* that swam expressed 5-HT2a and 5-HT7 receptor subtypes (Figure 4D1), whereas, A1 isolated from individuals that did not swim on the day of testing did not express any detectable 5-HT receptor subtype genes (Figure 4D2). This stands in contrast to C2 somata isolated from *Tritonia*, which consistently expresses both subtypes and which were consistently modulated by serotonin. It was the ability to unambiguously identity C2 and its homologs in different species (Lillvis et al., 2012) that allowed the mystery of neuromodulatory variability to be solved.

The cause of the fluctuations in gene expression in the *Pleurobranchaea* A1 neuron is still an open question. Although the factors that regulate gene expression have not been examined in this system, work from other systems suggests that regulation of gene expression is likely to be a common cause of neural circuit variation (Benowitz et al., 2018; Friedman et al., 2020). Temporal fluctuations in receptor gene expression may be representative of fluctuations of unknown regulatory factors, which may underlie the variability in genes, modulation, and behavior observed in *Pleurobranchaea*. This is consistent with a model that serotonin neuromodulation is responsible for creating the conditions that lead to the functional swim circuit. In the evolution of behavior and neural circuits, changes to the regulation of cellular expression of neuromodulatory receptors may be a more flexible point for natural selection to act on than other features of neurons (Katz, 2011, 2016).

## Conclusion

Neural circuits, like behaviors, exhibit individual variability. There are several challenges for neuroscience with regard to such variability. One is to distinguish between consequential and inconsequential individual differences in neuronal and synaptic properties. Some differences might underlie behavioral variability under normal conditions, whereas others might not have any effect on behavior unless the system is stressed (Onasch and Gjorgjieva, 2020). Understanding the effects of individual differences in neural circuit function might help in predicting and possibly ameliorating differential outcomes in injuries and diseases (Prabhakaran et al., 2008; Burke Quinlan et al., 2015; Dopfel et al., 2019). Heritable differences between individuals in neural circuits are the fodder for natural selection. Such differences may accumulate in a population if they have no effect on circuit function under normal conditions, but might be adaptive if conditions change.

A second challenge is to determine whether observed differences in neural circuits are caused by individual idiosyncrasies or whether they represent variations in the histories or states of the individuals. The ability to record from identified neurons for extended periods of time in gastropods has shown the extent to which the properties of individual neurons and synapses can vary within just a week; it is likely that over the course of a lifetime these properties could vary even further. Whether such intra-individual variability is commonly mistaken for inter-individual variability is an open question. Where possible, longitudinal studies of behavior and circuit properties will be necessary to determine whether this is the case.

## Author Contributions

All authors listed have made a substantial, direct, and intellectual contribution to the work, and approved it for publication.

## Conflict of Interest

The authors declare that the research was conducted in the absence of any commercial or financial relationships that could be construed as a potential conflict of interest.

## Publisher’s Note

All claims expressed in this article are solely those of the authors and do not necessarily represent those of their affiliated organizations, or those of the publisher, the editors and the reviewers. Any product that may be evaluated in this article, or claim that may be made by its manufacturer, is not guaranteed or endorsed by the publisher.
